# Fluorescent Protein-Expressing Neural Progenitor Cells as a Tool for Transplantation Studies

**DOI:** 10.1371/journal.pone.0099819

**Published:** 2014-06-16

**Authors:** Marco Skardelly, Eileen Hempel, Johannes Hirrlinger, Florian Wegner, Jürgen Meixensberger, Javorina Milosevic

**Affiliations:** 1 Translational Centre for Regenerative Medicine, University of Leipzig, Leipzig, Germany; 2 Department of Neurosurgery, University Hospital, Leipzig, Germany; 3 Carl-Ludwig-Institute for Physiology, Faculty of Medicine, University of Leipzig, Leipzig, Germany; 4 Department of Neurology, Hannover Medical School, Hannover, Germany; University of Nebraska Medical Center, United States of America

## Abstract

The purpose of this study was to generate quadruple fluorescent protein (QFP) transgenic mice as a source for QFP-expressing neural stem and progenitor cells (NSCs/NPCs) that could be utilized as a tool for transplantation research. When undifferentiated, these NSCs only express cyan fluorescent protein (CFP); however, upon neuronal differentiation, the cells express yellow fluorescent protein (YFP). During astrocytic differentiation, the cells express green fluorescent protein (GFP), and during oligodendrocytic differentiation, the cells express red fluorescent protein (DsRed). Using immunocytochemistry, immunoblotting, flow cytometry and electrophysiology, quadruple transgenic NPCs (Q-NPCs) and GFP-sorted NPCs were comprehensively characterized *in vitro*. Overall, the various transgenes did not significantly affect proliferation and differentiation of transgenic NPCs in comparison to wild-type NPCs. In contrast to a strong CFP and GFP expression *in vitro*, NPCs did not express YFP and dsRed either during proliferation or after differentiation *in vitro*. GFP-positive sorted NPCs, expressing GFP under the control of the human GFAP promoter, demonstrated a significant improvement in astroglial differentiation in comparison to GFP-negative sorted NPCs. In contrast to non-sorted and GFP-positive sorted NPCs, GFP-negative sorted NPCs demonstrated a high proportion of neuronal differentiation and proved to be functional *in vitro*. At 6 weeks after the intracerebroventricular transplantation of Q-NPCs into neonatal wild-type mice, CFP/DCX (doublecortin) double-positive transplanted cells were observed. The Q-NPCs did not express any other fluorescent proteins and did not mature into neuronal or glial cells. Although this model failed to visualize NPC differentiation *in vivo*, we determined that activation of the NPC glial fibrillary acid protein (GFAP) promoter, as indicated by GFP expression, can be used to separate neuronal and glial progenitors as a valuable tool for transplantation studies.

## Introduction

The utilization of the jellyfish green fluorescent protein (GFP) as a reporter of gene expression has opened new avenues of visualizing and mapping specific brain cell populations and neural microstructures [1]. Since its discovery, GFP and its derivatives such as enhanced GFP (EGFP), enhanced cyan FP (ECFP) and enhanced yellow FP (EYFP), have been increasingly used to mark neurons or glia in neural tissues. For this purpose, spectrally distinct fluorescent proteins (FPs) have been expressed under the control of neuronal and glial cell type-specific promoters. For example, the Thy1.2 promoter was employed to drive the expression of several FPs in neurons [2].

In this work, four different transgenic mice were crossed to obtain a quadruple transgenic mouse. To this end, we took advantage of the well-characterized promoters of the human glial fibrillary acidic protein (hGFAP), mouse proteolipid protein (PLP) and mouse Thy1.2 genes to target the expression of FPs in astrocytes, oligodendrocytes and neurons, respectively [3]–[5]. Endogenous neural stem cells (NSCs) and neural (neuronal or glial) progenitor cells (NPCs) of the adult brain have an intrinsic capacity to respond to brain disorders. A transgenic brain with the potential to generate multicolored brain cells from neural stem/progenitor cells may be a valuable model to track adult neurogenesis, including neuroblast migration, *in vivo* in both healthy and injured or diseased brains. In addition to other approaches that allow the *in vivo* visualization, identification, isolation and tracing of neural stem cells, such as immunophenotyping or infection with retro- or lentiviruses, this transgenic approach may be an excellent choice if well designed and functional.

In addition to the recruitment of endogenous NSCs and NPCs, recently popularized cell-based therapies for various CNS diseases and injuries rely primarily on the application of exogenous NPCs. Neuronal and glial progenitors have been successfully used in transplantation studies as a source of cells to replace damaged or lost brain cells because they give rise to neuronal and glial cell lineages [6]. Preliminary work has shown the propensity of NSCs to migrate toward areas affected by brain pathology; for example, they may be useful in anticancer treatment to deliver therapeutic proteins and genes to remove malignant brain tumors or may provide therapeutic improvement for neural repair through the delivery of growth factors, cytokines or neurotrophins [7], [8]. Various preclinical and clinical trials have utilized reliable *in vivo* tracking methods for transplanted cells. One option involves iron-labeling of NSCs, which enables their visualization and tracking by MRI [9]. However, several large hurdles must be overcome before such cell therapies can be applied to neural restoration. Specifically, the developmental stage of the progenitors needs to be clearly specified; furthermore, it is necessary to determine whether the outgoing phenotype of the transplanted cells fulfills the criteria for therapeutic effects and whether the transplanted cells make appropriate functional connections with target cells in situ.

The generation of multiple fluorescent protein-expressing neural stem cells would facilitate the translation of neural transplantation to future therapeutic treatments for various neuropathological conditions. In this study, we investigated the potency of QFP-expressing NPCs to serve as a tool in transplantation studies. We found that inactivity of the Thy1.2 and PLP promoters, both *in vitro* and *in vivo*, presents a major obstacle for the further preclinical use of these cells. Furthermore, we found that expression of GFAP in NPCs can be used to separate subpopulations such as neuronal and glial progenitor cells.

## Materials and Methods

### Mouse breeding

To obtain a transgenic mouse line expressing different fluorescent proteins in different types of brain cells, TgN(Thy1.2-EYFP) transgenic mice [10] whose neurons constitutively express yellow fluorescent protein (EYFP) were bred with double transgenic mice expressing GFP under the control of the human GFAP promoter [TgN(hGFAP-GFP)[11]] and DsRed under the control of the mouse PLP promoter [TgN(mPLP-DsRed) [10]], which resulted in EYFP expression in neurons, GFP expression in astrocytes and DsRed expression in oligodendrocytes. A transgenic mouse containing an enhanced cyan fluorescent protein gene under control of the chicken beta actin promoter TgN(CAG-ECFP) was purchased from JAX® Mice (The Jackson Laboratory, Maine, USA) and was bred with the other lines. The resulting transgenic quadruple transgenic mice were designated as Tg(CGYR).

To distinguish the different transgenic mice other than the Tg(CAG-ECFP) mice, which were visually identified by observation of the tail under a microscope, the mice were genotyped as previously described [10].

### Ethics Statement

Animal experiments were performed in accordance with the Policy on Ethics and the Policy on the Use of Animals in Neuroscience Research as indicated in the directive 2010/63/EU of the European Parliament and of the Council of the European Union on the protection of animals used for scientific purposes and were approved by the local authorities for care and use of laboratory animals (State of Saxony: internal reference number TVV 42/06). Animals were sacrificed under deep anaesthesia and all efforts were made to minimize suffering.

### Dissection and propagation of murine NPCs

Murine frontal (cortical) NPCs were dissected from transgenic murine brains at embryonic day 10.5 (E10.5). Based on the original transgenic mice (TgN(CAG-ECFP), the TgN(Thy1.2-EYFP), TgN(hGFAP-GFP/TgN(mPLP-DsRed) or Tg(CGYR)) cells were designated CFP-NPCs, YFP-NPCs, GFP/dsRED-NPCs or quadruple transgenic (Q)-NPCs. Pregnant mice were sacrificed. The presence of all four transgenes was confirmed for each fetal preparation microscopically and by PCR analysis. After dissection, fetal brain tissue samples were incubated in liberase-1-solution (200 µg/ml; Roche, Mannheim, Germany) containing 100 U/ml DNase I (Roche, Mannheim, Germany) and 1 mM MgCl_2_ for 30 min at 37°C. After being centrifuged at 200×g for 5 min, the samples were resuspended in Hank's Buffered Saline Solution (HBSS) containing 100 U/ml DNase I (Roche, Mannheim, Germany) and 1 mM MgCl_2_. Finally, the samples were homogenized by gentle trituration using a fire-polished Pasteur pipette. During the expansion phase, the cells were grown in a monolayer via plating onto polyornithine-fibronectin pre-coated cell culture dishes at a density of 10,000 cells/cm^2^. The cells were maintained in serum-free standard DMEM (high glucose)/F-12 medium mixture (1∶1) supplemented with 20 ng/ml epidermal growth factor (EGF), 20 ng/ml basic fibroblast growth factor (bFGF; both from PromoCell, Heidelberg, Germany) and 2% B–27 (without vitamin A, Invitrogen, Carlsbad, CA). The cells were passaged every 5 days by detachment with TrypLE™ (Invitrogen, Darmstadt, Germany). In passage 6 cell differentiation was initiated by omitting growth factors that support neuronal differentiation or by replacing the expansion medium with defined mitogen-free DMEM (high glucose)/F-12 medium supplemented with 10% fetal calf serum (FCS) to favor astroglial differentiation.

### Flow cytometry and fluorescence activating cell sorting (FACS)

For the flow cytometric analysis of FP expression in the different cell populations, the NPCs were enzymatically dissociated with TrypLE™, spun at 200×g for 5 min and resuspended in HBSS with 7-amino-actinomycin D (7-AAD, 0.1 µg/ml; Calbiochem, San Diego, CA) to exclude dead cells [12]. For each examination, 3×105 NPCs (105 cells/100 µl) were used. NPCs of wild type mice were used as a control to adjust the parameters for cytometric analysis. Only cells gated from the upper right quadrant (UR) from the forward scatter (FSC, x-axis) and side scatter (SSC, y-axis) dot plots, which were negative for 7-AAD incorporation, were used for further analyses. To enrich cultures of NPCs expressing GFP (GFP-positive cells), 1×106 hGFAP-GFP/mPLP-DsRed NPCs (P5) were harvested, and cells expressing high levels of GFP were sorted by FACS with FACSCanto (Becton Dickinson, Heidelberg, Germany) and BD FACSDiva™ software (BD Biosciences, San Jose, CA, USA) using a laser at a wavelength of 488 nm. After one passage (P6), the sorted cells were re-analyzed for GFP expression to estimate the percentage of GFP-positive and GFP-negative cells.

To characterize NPCs by flow cytometry, 5×10^5^ cells/200 µl were incubated in PBS with 0.1% bovine serum albumin (BSA) and the following primary antibodies for 30 min at 4°C in the dark to prevent internalization: anti-NCAM ((MEM-188)-PE 1∶100; Abcam, Cambridge, UK), PE/Cy7 anti-mouse CD24 (1∶100; BioLegend, San Diego, USA), anti-prominin-1-APC (1∶100; Miltenyi Biotec GmbH, Germany) and biotinylated anti-A2B5 antibody (1∶200; R&D Systems GmbH, Wiesbaden-Nordenstadt, Germany). The cells were then centrifuged (200×g) for 5 min, washed twice with 1 ml of PBS for 5 min, resuspended in 200 µl of PBS and re-incubated with streptavidin-conjugated Alexa Fluor 750 (1∶500; Life Technologies GmbH, Darmstadt, Germany) for a further 30 min at 4°C. After this incubation step, the cells were washed again with 1 ml of PBS for 5 min and centrifuged (200×g) for 5 min. Finally, the cells were resuspended in 300 µl of PBS with 7-AAD (0.1 µg/ml) and 0.1% BSA. The negative control samples were incubated according to the same protocol with the appropriate matched isotype control antibodies. Subsequently, the cells were analyzed by flow cytometry using lasers at 488 nm and 633 nm. Appropriate BD™ CompBead particles (BD Biosciences, San Jose, CA, USA) were used to optimize the fluorescence compensation for automated multicolor flow cytometric analysis. The sample flow rate during analysis did not exceed 1000 cells per second. All flow cytometric cell analyses were conducted using three independent cell populations that were then averaged (N = 3).

### Immunoblotting

Murine NPC extracts were prepared as previously described [13]. Basically, combined cytoplasmic and nuclear extracts were prepared in extraction buffer, and their concentrations were determined using the Bradford method with bovine serum albumin as a standard. Denatured proteins were resolved on sodium dodecyl sulfate (SDS)-polyacrylamide gels and transferred to Hybond-ECL nitrocellulose membranes (GE Healthcare, Freiburg, Germany) via semidry blotting. The membranes were stained with Ponceau S (Sigma) to confirm equal protein loading, transferred, blocked with 5% (wt/vol) nonfat dry milk in PBS-T (PBS, 0.1% (vol/vol) Tween 20) for 2 hours at room temperature and subsequently incubated with the desired primary antibody (diluted in PBS containing 5% non-fat milk and 0.1% Tween 20) overnight at 4°C with gentle agitation. The following antibodies were used: mouse monoclonal anti-actin (C4, 1∶2000; MP Biomedicals, Santa Ana, CA, USA), mouse monoclonal anti-PCNA antibody (1∶1000; Santa Cruz, Heidelberg, Germany) and horseradish peroxidase-coupled secondary antibodies (Pierce, Rockford, IL, USA). Chemiluminescence detection was performed by incubating the membranes with SuperSignal-Dura substrate (Pierce) followed by analysis on a CCD cooling camera (Fuji LAS-1000plus). The chemiluminescence was quantified using 2-dimensional densitometry software AIDA (Raytest Isotopenmeb, Straubenhardt, Germany). All immunoblots were performed in duplicates of three independent cell populations and averaged (N = 3).

### Immunofluorescence

Murine NPCs under expansion were seeded onto sterile glass cover slips, fixed with 4% paraformaldehyde in PBS for 10 min at room temperature and washed with PBS. Subsequently, the cells were counterstained with the DNA-binding dye, 4'-6-diamidino-2-phenylindole (DAPI, 2 µg/ml in PBS), followed by incubation in blocking buffer. For immunofluorescence, the following primary antibodies were used: mouse monoclonal anti-nestin (1∶400; Pharmingen, San Diego, CA, USA), rabbit polyclonal anti-β-tubulin III (Tuj1 1∶1000; Covance, Freiburg, Germany), rabbit polyclonal anti-Ki67 antigen (1∶500; Novocastra Laboratories Ltd, Newcastle upon Tyne, UK), mouse monoclonal anti-GFAP (1∶400; Chemicon International, Hampshire, UK), rabbit polyclonal anti-microtubule associated protein-2 (MAP2ab, 1∶500; Chemicon International, Hampshire, UK), mouse monoclonal anti-oligodendrocyte marker O4 (1∶500; R&D Systems, Minneapolis, MN, USA), goat polyclonal anti-doublecortin (1∶200; Santa Cruz Biotechnology, Inc., Santa Cruz, CA, USA), goat polyclonal anti-GFP (1∶2000; Acris antibodies, San Diego, CA, USA) and rabbit polyclonal anti-Sox2 (1∶1000; Chemicon International, Hampshire, UK). Finally, fluorescent secondary antibodies conjugated to Alexa Fluor® 488 or Alexa Fluor® 594 (1∶500; Life Technologies GmbH, Darmstadt, Eugene, USA) or streptavidin-conjugated secondary antibodies were used. Coverslips were mounted onto glass slides and examined using a confocal laser-scanning microscope (LSM 510, Zeiss, Oberkochen, Germany) at excitation wavelengths of 594 nm (helium/neon, red Alexa 594 immunofluorescence) and 488 nm (argon, yellow-green Alexa 488 immunofluorescence). Alternatively, the glass slides were examined under a fluorescence microscope (Zeiss Axiovert 200). The acquisition of the immunostained cells was performed using the image-analysis software AxioVision 4 (Carl Zeiss AG, Jena, Germany). All stained cells were counted in three randomly selected fields (N = 3) of three independent cell populations and averaged (N = 3).

### Electrophysiology

Whole-cell patch-clamp recordings of voltage-gated ion channels were performed in the voltage- or current-clamp mode (holding potential −70 mV) at room temperature using an EPC-9 amplifier and PulseFit software (HEKA, Lambrecht, Germany) as previously described [14]. The external bath solution contained (in mM): 142 NaCl, 1 CaCl_2_, 8 KCl, 6 MgCl_2_, 10 glucose and 10 HEPES (pH 7.4; 320 mOsm). Micropipettes were formed from thin-walled borosilicate glass (BioMedical Instruments, Zöllnitz, Germany) with a Flaming Brown P-97 electrode puller (Sutter Instrument Co., Novato, CA, USA) and a Micro Forge (Narishige, Tokyo, Japan). The electrodes had resistances of 2–4 M when filled with the internal solution containing (in mM): 153 KCl, 1 MgCl2, 10 HEPES, 5 EGTA, and 2 MgATP (pH 7.3; 305 mOsm).

Whole cell currents were low-pass-filtered at 1–5 kHz, digitized at 10 kHz and analyzed using PulseFit (HEKA, Lambrecht, Germany) and GraphPad Prism version 4.00 for Windows (GraphPad Software, San Diego, CA, USA). The numerical data of all experiments were expressed as the mean ± SEM, with the statistical significance determined using Student's t-test (two-tailed, unpaired; p<0.05).

### Intra-cerebroventricular transplantation of Q-NPCs

In total, 10^5^ *in vitro*-expanded Q-NPCs (P3) were transplanted into each lateral ventricle of neonatal C57BL/6 mice on the day of birth. In particular, neonatal C57BL/6 mice were anesthetized on ice, and 2 µl of the Q-NPC cell suspension was injected into the lateral ventricle using a fire-polished glass micropipette. The cells were suspended in PBS that contained 0.05% (w/v) trypan blue for direct visualization of the transplantation process. After 6 weeks of engraftment, the mice were transcardially perfused using 4% paraformaldehyde in PBS. The dissected brains were postfixed at 4°C until used, and 50 µm-thick brain tissue sections (8–12 slices) were chosen for analysis. The sections were immunostained with the antibodies and analyzed by confocal laser scanning microscopy as described in the immunofluorescence section.

### Statistics

Student's *t*-test was performed for comparison of significant differences between the two groups. Normally distributed data were statistically evaluated using an appropriate analysis of variance (ANOVA) followed by Bonferroni's test for multiple comparisons (SigmaStat 3.5 software package, Jandel Corp., San Rafael, CA). A non-parametric test (Kruskal-Wallis) was used when the data were not normally distributed. If the result of the Kruskal-Wallis test was significant (P<0.05), Dunn's test for multiple comparisons, computed on ranks rather than data, was performed. Significance was defined as p<0.05. All data are expressed as the mean ± SEM.

## Results

To evaluate the potency of single and multiple fluorescent NPCs as tools for transplantation studies, we first investigated the *in vitro* expression and stability of fluorophores during passaging and the potential to separate subpopulations by FACS. Next, we characterized the developmental stage of NPCs *in vitro* by multicolor flow cytometry. We explored whether single and multiple fluorescent transgenes interfere with the proliferation and differentiation capacity of NPCs *in vitro* by immunofluorescence and immunoblotting. Subsequently, we investigated the electrophysiological properties of GFP-positive and GFP-negative NPCs *in vitro* by whole-patch-clamp recordings. Finally, we evaluated the presence and *in vivo* differentiation of intra-cerebroventricular transplanted transgenic Q-NPCs 6 weeks after transplantation in neonatal mice.

### Transgene expression in NPCs

The percentage and extent of *in vitro* fluorophore expression in single and quadruple transgenic NPCs were evaluated by flow cytometry during passaging. Moreover, the ability to separate NPC subpopulations using the GFP expression of TgN(hGFAP-GFP) was evaluated by FACS.

**Figure 1:**
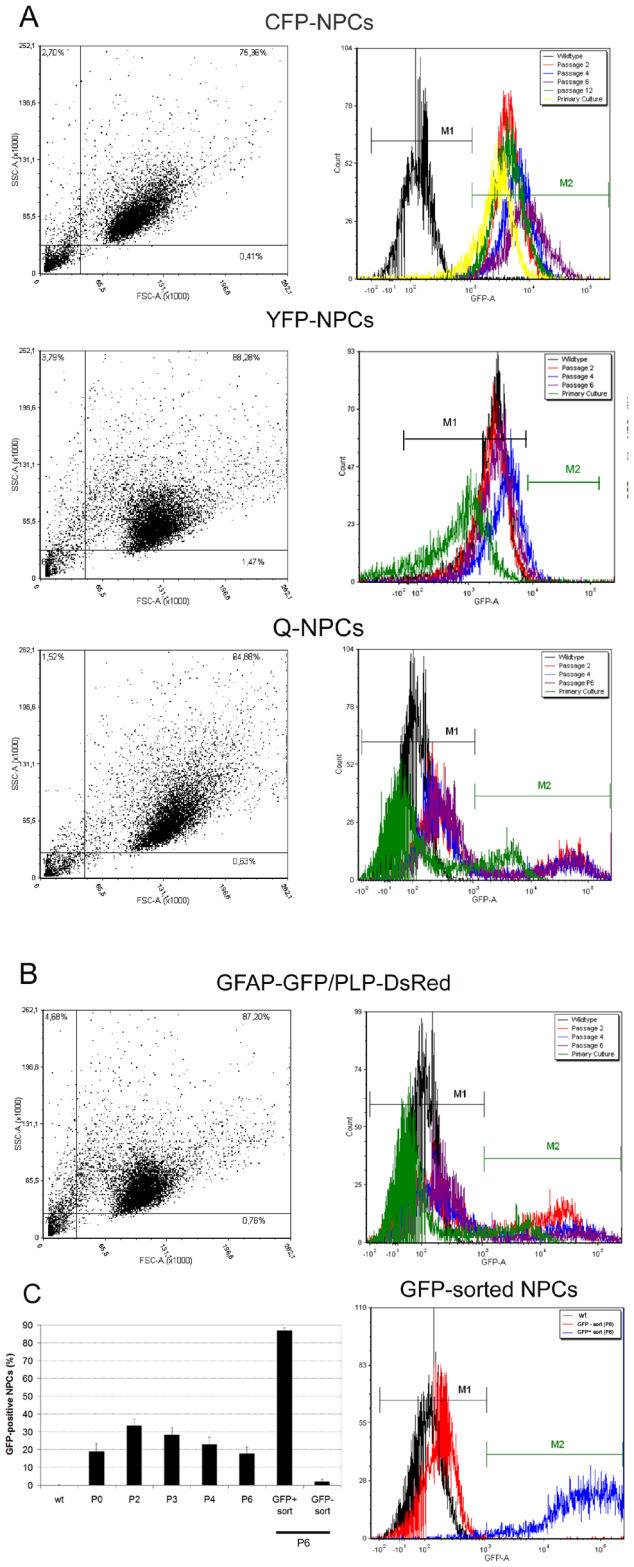
Flow cytometric analysis of the long-term expression of FP in murine NPCs. (A) Panel showing dot plots of the respective NPC populations (CFP, YFP and QFP) on the left and the histograms of FP expression (M2) in comparison to the wild type (M1) during passaging on the right. The primary cultures and the first several passages (P2 to P12) of expanded NPCs dissected from Tg(CAG-ECFP) (shown as CFP-NPCs), TgN(Thy1.2-EYFP) (shown as YFP-NPCs) and quadruple Tg(CGYR) transgenic embryos (Q-NPCs) are shown. Panel (B) shows the cell population dot plot and the GFP expression histogram in the total population of NPCs prepared from double transgenic TgN(hGFAP-GFP/mPLP-DsRed) mice over the passages (P2-P6). (C) A non-significant reduction in GFP-positive cells from P2 to P6 is illustrated on the left, and enrichment of the NPC population positive for GFP after FACS sorting is shown on the right.

More than 75% of all transgenic cells examined in their proliferation phase after detachment appeared to be viable as shown in FSC x SSC dot plots (Fig. 1A). Through several passages, CFP expression did not significantly change, but it changed significantly in comparison to the primary culture (N = 3, p<0.05). In the primary culture (P0), 86.2±0.7% of the vital cells were CFP-positive; however, 96.4±1.3% were CFP-positive at passage 2 (P2) and 96.6±1.4% were CFP-positive at passage 12 (P12). During expansion, almost all YFP-NPCs remained non-fluorescent (99.2±0.1 in P2 and 98.8±0.7 in P6) (Fig. 1A). Quadruple transgenic NPCs (Q-NPCs) displayed a stable amount of GFP-positive cells (approximately 30%) over passaging. A significant alteration in the percentage of GFP-positive cells (N = 3; p = 0.003; one way ANOVA) was only observed when comparing the primary culture (P0) (9.8±3.7%) with one of the other passages (Fig. 1A). GFP-NPCs dissected and *in vitro* expanded from double-transgenic TgN(hGFAP-GFP/mPLP-DsRed) mice exhibited a significant reduction in GFP fluorescence, with the proportion of GFP-positive cells decreasing from 33.5±3.8% in P2 to 17.6±3.9% in P6 (Fig. 1B). Post-sorting flow cytometric assessment of the cells confirmed that 86.9±1.5% of the cells were GFP-positive. One-way ANOVA followed by Bonferroni's post-hoc test revealed a significant difference in GFP expression in P6 NPCs relative to GFP-positive-sorted NPCs (also P6) (N = 3, p<0.05, Fig. 1C).

Overall, only TgN(CAG-ECFP) was expressed in almost all cells and was stable during 12 passages. In contrast, TgN(hGFAP-GFP) expression decreased from only 1/3 (P2) to 1/6 (P6) of all cells but separated the NPCs into two different subpopulations.

### Flow cytometric characterization of NPCs

Although the NPCs harvested and cultivated under the prescribed conditions were already characterized in detail, we further explored the nature of the cells using multicolor flow cytometry.

**Figure 2:**
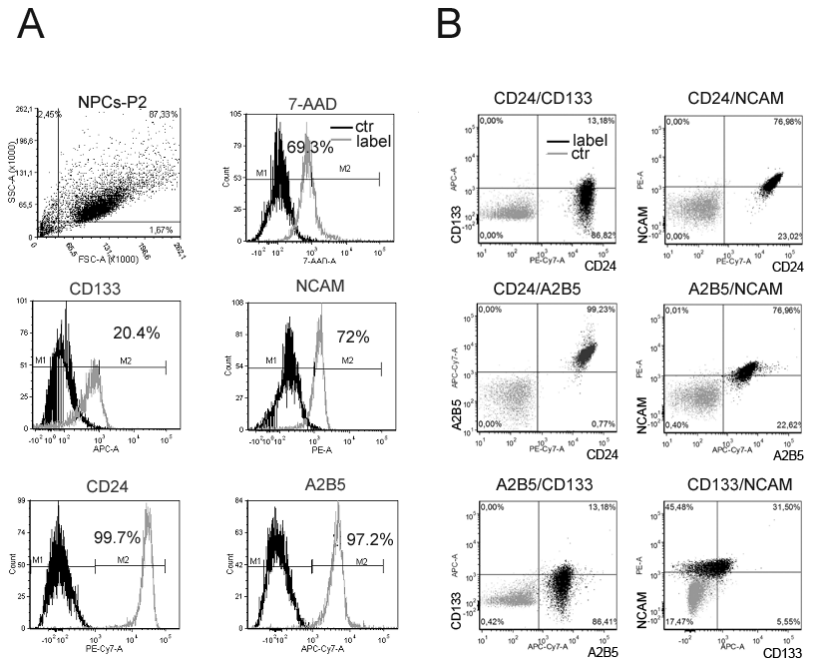
Flow cytometric analysis of surface biomarker expression by NPCs. (A) Dot plot of gated living P2 NPCs followed by respective histograms of NPCs stained for the cell surface markers CD133, NCAM, CD24 and A2B5. Gray lines represent positive staining, and black lines show isotype controls. Percentages indicate the number of positive cells (M2) within each population of stained cells in comparison to negative cells (M1). Most analyzed NPCs excluded 7-AAD viability staining solution and showed different percentages of marker expression. (B) FACS dot plots showing, in percentages, the combination of various surface neural progenitor/stem cell markers as indicated above each dot plot.

Expanded NPCs were comprehensively characterized *in vitro*. Using flow cytometry, dead cells were gated out as they emerged 7-AAD positive. Further analysis was performed on 7-AAD-negative (living) cells (69.3±1.4%, Fig. 2A). In total, 20.4±2.0% of NPCs positively stained for CD133 (prominin-1), a stem cell marker. As shown in the histogram plot (Fig. 2A), 71.5±5.7% of NPCs exhibited the surface marker NCAM; however, the remaining 28.5±5.8% appeared negative for NCAM. Virtually all of the NPCs (99.7±0.1%) were positive for CD24, which is another surface biomarker relevant for neural lineage stem cell fate determination. Similarly, 97.2±0.8% of the NPCs expressed the neural progenitor marker A2B5. As shown in Fig. 2B, 99.2% of the cells were CD24-positive and A2B5-positive. The following four subtypes of NPCs were identified by simultaneous staining of CD24, CD133, A2B5 and NCAM: A) CD24^+^/A2B5^+^/NCAM^+^/CD133^+^ (45.5%), B) CD24^+^/A2B5^+^/NCAM^+^/CD133^−^ (31.5%), C) CD24^+^/A2B5^+^/NCAM^−^/CD133^+^ (5.5%) and D) CD24^+^/A2B5^+^/NCAM^−^/CD133^−^ (17.5%).

In summary, we identified four subtypes of NPCs. All of the cells expressed CD24 and A2B5 and were differentiated by the presence or absence of CD133 and NCAM expression.

### Proliferation and differentiation of fluorescing NPCs

We investigated the influence of single and quadruple transgenes on proliferating NPCs by assessing the immunofluorescence of the stem cell markers Nestin and Sox2 and the proliferation markers Ki67 and PCNA via immunoblotting. The differentiation of the transgenic NPCs was evaluated by expression of the glial markers GFAP and O4 in addition to the early and late neuronal markers Tuj-1 and MAP2. Furthermore, the two subpopulations of expressing and non-expressing TgN(hGFAP-GFP) cells were characterized regarding their tendency to differentiate towards astrocytic or neuronal lineages.

The proportion of CFP (98±1%) and GFP expression (18±6%) remained stable during differentiation of transgenic NPCs. YFP and dsRed expression was neither observed during proliferation nor after differentiation of transgenic NPCs.

**Figure 3:**
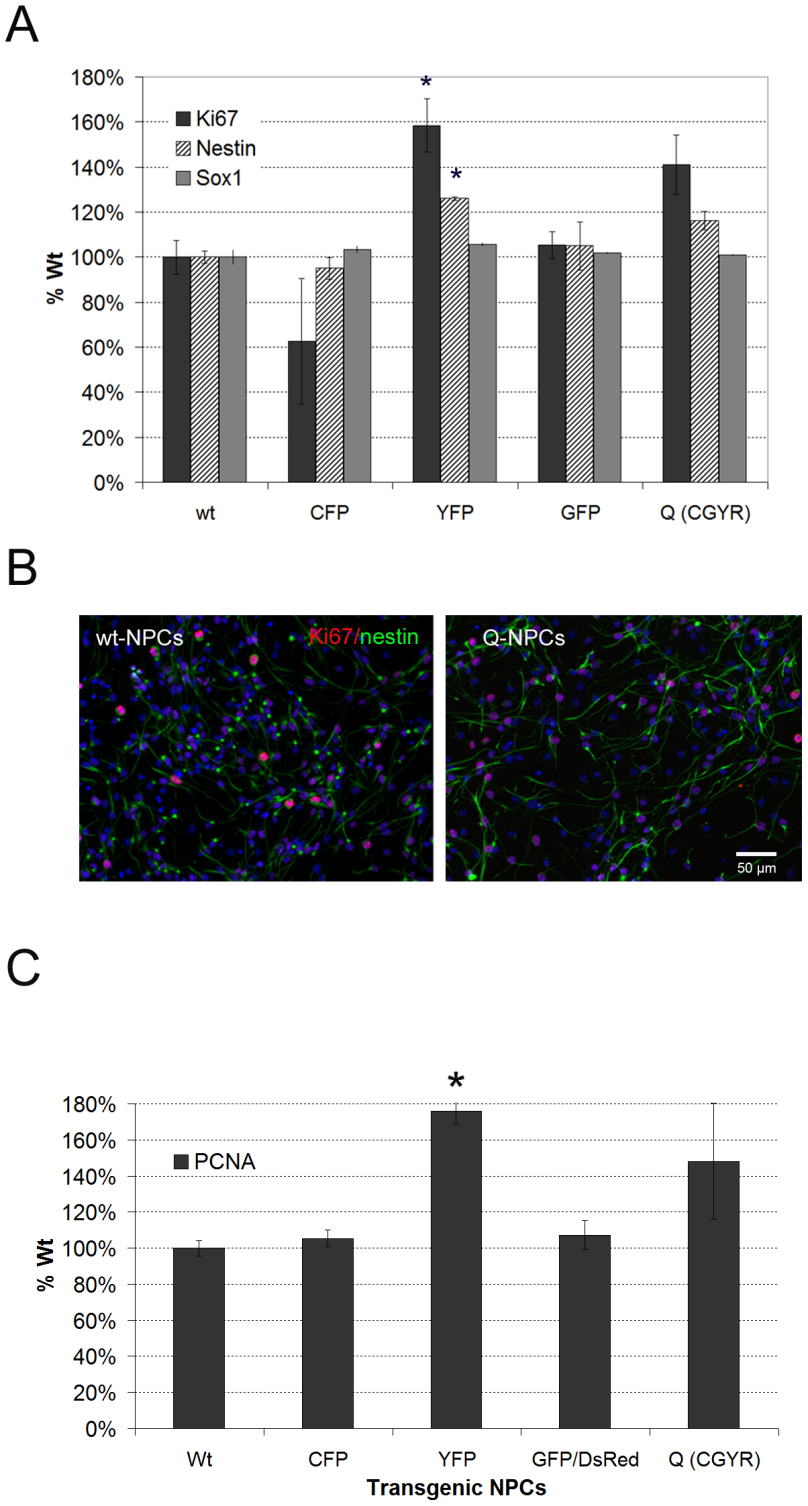
Immunocytochemistry of expanded transgenic NPCs. (A) Relative proportion of Ki67-, nestin- and Sox2-positive cells in expanded NPCs comprising various transgenic constructs as designated in comparison to wild type-NPCs (X-axis, mean with SEM, N = 6). *p<0.05 vs. wild type. (B) Representative immunofluorescent labeling with Ki67 (red) and nestin (green) in quadruple transgenic NPCs (Q-NPCs) versus wild type NPCs. (C) Column bar graph of immunoblotting showing the proliferation capacity (PCNA) of various transgenic NPCs (mean, SEM, N = 6). *p<0.05 vs. wild type (Dunn's method).

One-way ANOVA revealed a significant increase in the proliferative capacity of NPCs carrying different transgenes (X-axis, Figure 3A) (N = 3, p<0.001). Thus, early passaged (P6) YFP-NPCs displayed a 58±12% higher number of Ki67-positive cells than wild type-NPCs. In the same cells, nestin was expressed in 26±1% more cells relative to wt-NPCs as determined by Bonferroni's post-hoc test (N = 3, p<0.05). Although Q-NPCs also demonstrated an increase in Ki67- (41±12%) and nestin-positive cells (16±6%), the difference was not statistically significant. The expression pattern of Ki67/nestin was similar in the remaining transgenic cells (Figure 3B). In all the transgenic cells, SOX2 was expressed at approximately the same level as in wild type-NPCs (p = 0.13). Although the PCNA proliferation marker tended to show increased expression in Q-NPCs, an overall ANOVA on ranks followed by Dunn's post-hoc test only revealed a significant increase in NPCs carrying the YFP transgene alone compared to wild type cells (76±7% increase over wild type; Fig. 3C) (N = 6, p<0.05).

**Figure 4:**
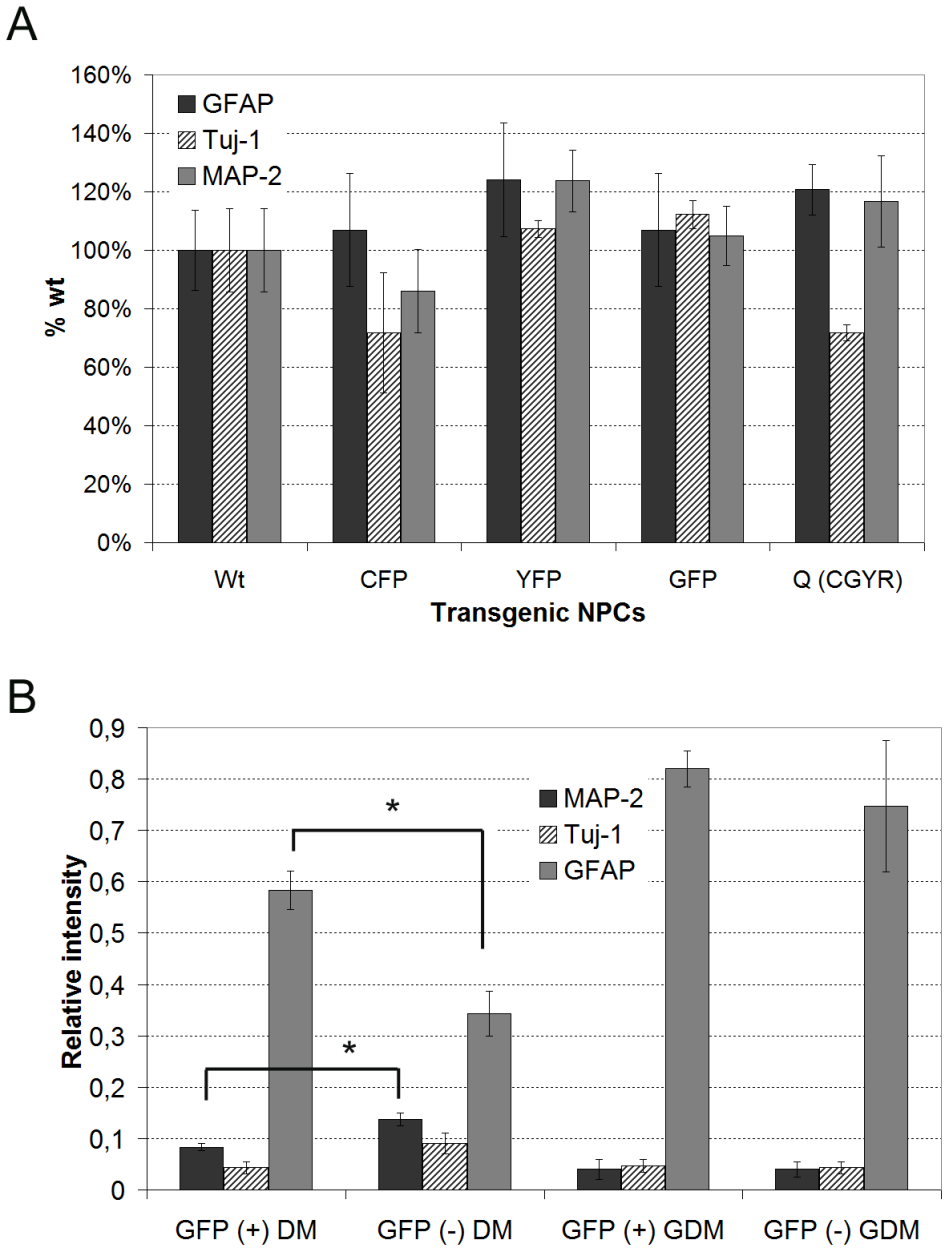
Differentiation of NPCs containing various transgenes. (A) The relative proportion of GFAP-, Tuj-1- and MAP2-positive cells in differentiated NPCs carrying different transgenes examined at P6 in comparison to wild-type NPCs is displayed in a column bar graph with the mean and SEM (N = 3). (B) Markers of glial (GFAP) and neuronal (Tuj-1 and MAP2) differentiation examined by immunofluorescence in GFP-sorted NPCs (positive and negative) upon differentiation for 2 weeks in two different media. DM =  common differentiation medium, GDM  =  glial differentiation medium (mean, SEM, N = 3). *p<0.05 (*t*-test).

The number of cells positive for astrocytic or neuronal differentiation was not altered in different transgenic NPCs in comparison to wild type NPCs differentiated in common differentiation medium (DM, N = 3; Fig. 4A). Overall, the various transgenes did not significantly affect astrocytic (GFAP, p>0.05), early neuronal (Tuj-1, p>0.05) and late neuronal (MAP2, p = 0.041, n.s. because of correction for multiple comparisons) differentiation.

The comparison of GFP-positive and GFP-negative sorted NPCs during early neurogenesis in common DM showed a slight but non-significant increase in GFP-negative sorted NPCs. A significant increase in MAP2, a marker of mature neurons, was detectable in GFP-negative vs. GFP-positive sorted NPCs (p<0.05, N = 3, Fig. 4B, left bar charts). GFP-positive sorted cells demonstrated a significant improvement in astroglial differentiation in comparison to GFP-negative sorted NPCs when both were differentiated in common DM (p<0.05, N = 3; Fig. 4B left bar charts). In general, Tuj-1 and MAP2 neuronal markers exhibited reduced expression in glial differentiation medium (GDM, see Materials and Methods section) versus common DM; however, GFAP showed apparently increased expression in both types of sorted NPCs in GDM (Fig. 4B). However, the changes in GFAP, Tuj-1 and MAP2 expression between GFP-positive and GFP-negative sorted NPCs that differentiated in GDM were not significantly different (Fig. 4B, right bar charts).

In summary, YFP and dsRed were not expressed under proliferation and differentiation of NPCs carrying TgN(Thy1.2-EYFP) or TgN(hGFAP-GFP/mPLP-DsRed). The transgenes showed no remarkable effect on the proliferation and differentiation of transgenic NPCs in comparison to wild-type cells other than an induction of proliferation (Ki67 and PCNA) in NPCs carrying TgN(Thy1.2-EYFP). Moreover, TgN(hGFAP-GFP) NPCs demonstrated significantly higher differentiation towards the astrocytic lineage through significantly elevated expression of GFAP and reduced expression of MAP2 in comparison to non-expressing TgN(hGFAP-GFP) NPCs.

### Electrophysiological properties of sorted NPCs

To further characterize the subpopulations of expressing and non-expressing TgN(hGFAP-GFP) NPCs, we investigated the electrophysiological properties of mouse frontal GFP-positive and GFP-negative NPCs during proliferation and after differentiation for 2 weeks *in vitro*.

**Figure 5:**
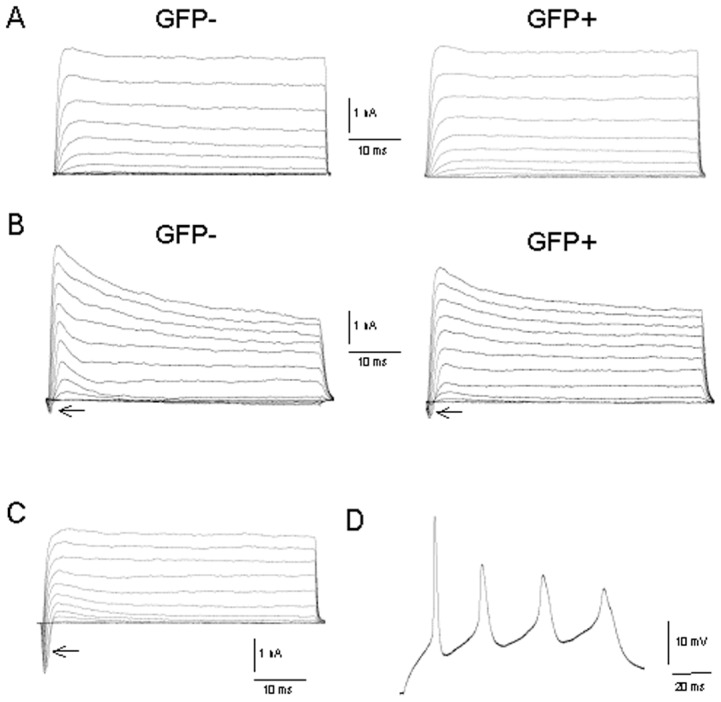
Voltage-gated ion currents of mouse frontal NPCs during expansion and after differentiation *in vitro*. A–B, Recordings were obtained in whole-cell voltage-clamp mode by stepwise depolarization with increasing amplitude from the holding potential of −70 to 50 mV in steps of 10 mV. During proliferation, the GFP-negative (left) and GFP-positive (right) NPCs (P6) displayed similar potassium outward current amplitudes (A). After differentiation with differentiation medium (DM) containing 10% FCS (GDM), sodium inward currents (arrows) were also detectable in both cell types (B). C, Recordings from a neuronal GFP-negative cell (2 weeks of DM-differentiation) with large sodium inward currents (arrow). D, The same cell as in C fired repetitive action potentials in response to a depolarizing current pulse (30 pA) at a holding potential of −72 mV in whole-cell current-clamp mode.

Whole-cell recordings were performed in the voltage-clamp mode to measure voltage-gated potassium and sodium currents (Figure 5, A–C). In the current-clamp mode, we evaluated the capacity of the NPCs to generate action potentials in response to depolarizing current pulses (Figure 5, D). The peak sodium currents of GFP-positive and -negative cells increased significantly during maturation (Figure 5, A–C, Table 1). However, action potentials in response to depolarizing current pulses were elicited only in GFP-negative NPCs differentiated using the common differentiation medium (DM) (D, N = 7/13). After differentiation with DM, the GFP-positive cells were not recordable, most likely because of membrane disintegration. GFP-positive and GFP-negative NPCs that differentiated in DM containing 10% FCS (GDM), were not able to fire single action potentials because of insufficient sodium inward currents. The potassium outward currents, the resting membrane potential and membrane capacitance were not significantly different; however, the input resistance of the NPCs after differentiation with DM showed a significant increase (Table 1) similar to the values in maturing human NPCs (Wegner et al., 2009).

**Table 1:** Functional properties of mouse NPCs during expansion and after differentiation for 2 weeks *in vitro*.

Functional properties of NPCs	Expanded GFP− (n = 12)	Expanded GFP+ (n = 9)	Differentiated GFP− (DM3, n = 5)	Differentiated GFP+ (DM3, n = 5)	Differentiated GFP− (DM, n = 13)
Peak Na^+^ current	−51±17 pA	−25±13 pA	−221±79 pA**	−305±105 pA**	−575±167 pA**
Peak Na^+^ current/pF	−4.3±1.5 pA/pF	−2.2±1.6 pA/pF	−22.3±9.2 pA/pF**	−30.8±11.3 pA/pF**	−59.0±19.6 pA/pF*
Peak K^+^ current	2598±427 pA	2339±581 pA	2705±325 pA	2342±456 pA	2684±162 pA
Peak K^+^ current/pF	235±49 pA/pF	270±107 pA/pF	236±81 pA/pF	227±101 pA/pF	299±49 pA/pF
Membrane capacitance	13.2±1.9 pF	15.9±4.9 pF	16.6±4.3 pF	15.0±4.5 pF	10.6±1.0 pF
Membrane potential	−43.5±6.5 mV	−54.6±4.3 mV	−54.0±6.6 mV	−38.5±11.8 mV	−37.3±3.4 mV
Input resistance	190±53 MΩ	94±25 MΩ	116±19 MΩ	135±18 MΩ	568±129 MΩ*

Voltage-gated currents and passive membrane properties of GFP-positive and GFP-negative cells are shown during proliferation and after differentiation with DM3. Note that only GFP-negative NPCs were recordable after differentiation with DM for 2 weeks. All data are shown as the means ± SEM, and a statistical comparison was calculated for differentiated NPCs and the respective GFP-positive or GFP-negative cells during expansion (*p<0.05, **p<0.01, Student's t-test).

Although both GFP-positive and GFP-negative TgN(hGFAP-GFP) NPCs showed increasing peak sodium currents during maturation, only GFP-positive cells demonstrated action potentials after differentiation with neuronal differentiation medium.

### Intra-cerebroventricular transplantation of Q-NPCs

We investigated the survival and differentiation of intracerebroventricular transplanted Q-NPCs by immunohistochemistry. Q-NPCs were transplanted into the lateral ventricle of neonatal wild type-mice. The cells were allowed to migrate, differentiate and mature for 6 weeks without immunosuppression.

**Figure 6:**
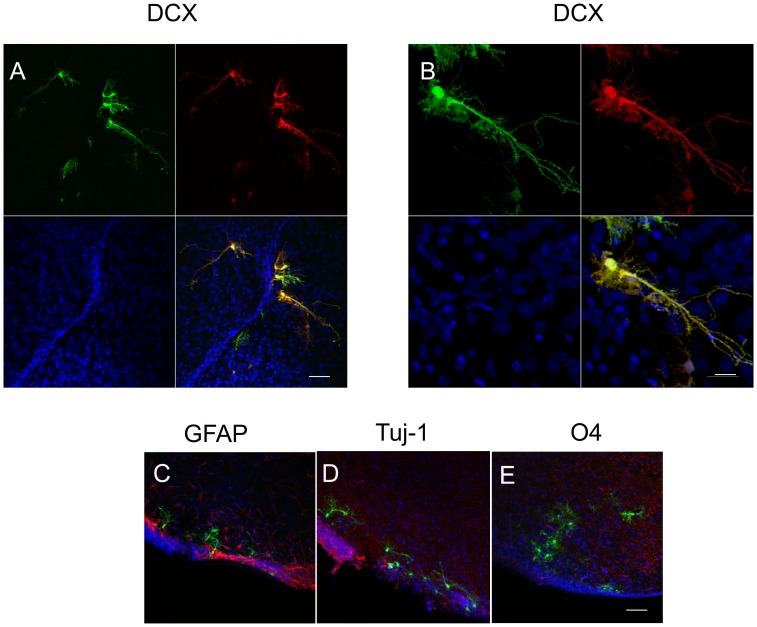
Q-NPCs give rise to doublecortin-positive (DCX-positive) cells in vivo. Laser scanning images of *in vivo* differentiation and maturation of quadruple transgenic neuronal precursors (Q-NPCs) following transplantation into the lateral ventricle of newborn wild-type mice. (A, B) At 6 weeks after intraventricular transplantation, a portion of the transplanted cells (GFP-positive in green, additionally stained with an antibody against GFP) generated DCX-positive cells (red) but remained negative after staining (red) for glial marker GFAP (C), neuronal marker Tuj-1 (D) and oligodendrocyte marker O4 (E). DAPI staining is shown in blue. Scale bar (A, C–E) 50 µm, (B) 20 µm.

Various transplanted cells survived, migrated and integrated into the subventricular zone expressing CFP but not the other fluorochromes. Subsequently, the cells were examined immunohistochemically. Brain slices were stained for GFP to increase the CFP signal in expressing Q-NPCs. In parallel, the expression of other markers was investigated. As presented in Figure 6, the transplanted cells survived and exhibited a moderate CFP signal (green) that merged with the red doublecortin (DCX) signal in the majority of GFP-positive NPCs (Fig. 6A, B). The transplanted cells failed to demonstrate immunoreactivity to antibodies against GFAP, Tuj-1 and O4 (Fig. 6, C–E).

Q-NPCs survived 6 weeks after transplantation, migrated into the subventricular zone and integrated as determined by their formation of multiple dendrites, but they only expressed CFP and failed to show immunoreactivity to any differentiation antibodies other than DCX.

In summary, the proliferation and differentiation of the quadruple transgenic NPCs were not remarkably different from those of wild-type NPCs. The transgenic expression of TgN(CAG-ECFP) and TgN(hGFAP-GFP) was approximately 100% and 30%, respectively. Other fluorophores were not expressed *in vitro*. Although Q-NPCs survived, migrated and integrated after transplantation *in vivo*, they only presented expression of TgN(CAG-ECFP) and DCX immunoreactivity. Moreover, TgN(hGFAP-GFP) separated two different subpopulations of NPCs based on the presence or absence of GFP expression. After differentiation, the GFP-positive and GFP-negative NPCs presented morphological and functional characteristics of glial cells and neurons, respectively.

## Discussion

Transgenic labels that allow the visualization and tracking of specific populations of brain cells represent valuable tools for neurological research. In the present study, four different transgenic mice were crossed to obtain a quadruple transgenic mouse useful for the preparation of quadruple transgenic neural progenitor cells (Q-NPCs). These transgenic NPCs are useful for neural stem/progenitor transplantation research because they enable the tracing of NPC fate *in vivo*. Exogenous neural stem/progenitor cells represent a promising source of cells for cell replacement therapy in various neurological disorders such as Parkinson's disease, Huntington's disease, stroke, traumatic brain injury, spinal cord injury, motor neuronopathies, myelin disorders, lysosomal storage disorders and other enzymatic deficiency diseases [15]. In addition, NPCs have been proposed as a new and promising treatment modality in various brain pathologies including malignant brain tumors [16]. Molecular imaging is the most useful technique for the successful delivery, safe monitoring and spatiotemporal kinetic analysis of NPCs following transplantation [17].

### Transgene expressions of Q-NPCs

In this study, the quadruple transgenic NPCs generated were designed to trace the maturation of transplanted NPCs in different colors *in vivo*. First, the activities of the corresponding promoters of Q-NPCs were tested *in vitro*. While most of the viable NPCs dissected from the CFP mice exhibited CFP expression (approximately 97% in passage 12), GFP expression in proliferating NPCs from double transgenic TgN(hGFAP-GFP/mPLP-DsRed) mice did not exceed 33%, which suggests that the hGFAP promoter is only active in a subpopulation of NPCs [18]. GFAP is highly expressed in differentiated astrocytes but is also known to be expressed in radial glial cells (Type B cells), which are neural stem cells in the subventricular zone (SVZ) *in vivo* [19]. Flow cytometric analysis and immunofluorescence further revealed the complete absence of YFP and DsRed expression in proliferating NPCs but also after differentiation, suggesting a lack of Thy1.2 and PLP promoter activity [2], [20]. These findings indicate virtually no far-reaching neuronal and oligodendroglial maturation after differentiation of NPCs or at least no activation of both promoters under the applied *in vitro* conditions. This finding was not unexpected for Thy1.2 because it is expressed only in primarily fairly mature neurons [21], [22]; however, this result is intriguing for PLP because PLP is also expressed in NPCs *in vivo* [23].

### Assessment of multipotency of Q-NPCs

Immunocytochemical analysis of Q-NPCs revealed that in comparison to wild type NPCs, the expression of other NPC markers such as SOX2 and nestin and the proliferation markers Ki67 and PCNA remained unchanged during cell expansion [24], [25]. The differentiation potential as determined by immunocytochemistry *in vitro* showed unaltered expression of neuronal (Tuj-1, MAP2) and glial (GFAP) markers. Altogether, our results indicate that the multipotency of quadruple transgenic NPCs remains unaffected, which suggests that the cells will retain their capability to differentiate into all three types of neural cells upon transplantation. However, in our investigation, we were unable to observe the expected *in vivo* maturation of Q-NPCs, although 6 weeks following intra-cerebroventricular transplantation of Q-NPCs, the CFP-positive cells survived and exhibited neuronal commitment by expressing the neuroblast marker doublecortin (DCX). Surprisingly, the cells did not show Tuj-1 immunoreactivity, which is usually present in immature DCX-positive neurons [26], [27], and no staining was observed for GFAP, which is an indicator of NPC differentiation and maturation *in vivo*. One potential concern is that the transgenes may not have been active in the quadruple transgenic fetuses used for the preparations. However, this concern appears unlikely because the presence of the transgenes was confirmed by PCR, and the expression patterns of the transgenes are known to be very stable [10], [11]. This notion is supported by the finding of strong expression of FPs in neurons, astrocytes and oligodendrocytes similar to that of the single transgenic lines in this novel quadruple transgenic line. However, why cells that were multipotent and prone to regular differentiation *in vitro* failed to develop brain cell phenotypes *in vivo* remains unclear. A logical explanation is that negative environmental cues prevented full differentiation of the transplanted cells; however, this explanation is unlikely given the plasticity of NPCs and that the cells were transplanted into the ventricular brain region, which facilitates widespread dissemination, including to neurogenic regions [28], [29].

### Characterization of NPC subpopulations

Proliferating NPCs that were cultured under the applied *in vitro* conditions were comprehensively analyzed *in vitro*. Flow cytometric fractionation for YFP revealed virtually no expression, highlighting the undifferentiated state of the Q-NPCs. Expression of molecular markers associated with neural stem/progenitor cells such as prominin-1 (CD133), NCAM, CD24 and A2B5 confirms this finding. An increasing body of evidence connects CD133 expression and the maintenance of an immature cell phenotype, and CD133 has thus been used to define and purify self-renewing cell populations in different tissues [30]. CD133 is found in human NPCs and in adult neural progenitors [31], [32]; however, it is rarely expressed by radial glial cells [33]. Adhesion molecules such as neural cell adhesion molecule (NCAM) are expressed on the surface of NPCs, and NCAM is involved in the migration of newborn cells in the adult hippocampus and subventricular zone (SVZ) [34], [35]. The role of adhesion molecules in transplantation studies for the incorporation and functional integration of grafted cells should not be neglected. The most strongly expressed surface markers included CD24 and A2B5, with virtually all cells being double-positive. CD24 is known to be an NPC neural stem marker [36] related to neuronal development [37], [38]. In contrast, A2B5 is known to predominantly label glial progenitors [39] but has also been observed in neuronally committed NPCs [40]. Furthermore, the different steps of cell maturation from omnipotent stem cells to differentiated cells represent a continuum of increasing and decreasing expression of myriad proteins. Depending on the extrinsic signals, which are less consistent *in vitro* than *in vivo* because variability in cell culture conditions, very similar cells may exhibit different surface protein patterns. In this study, we identified four subtypes of NPCs, which were characterized by the presence or lack of NCAM and CD133 expression. Further studies have to be done to investigate the impact of NPCs' subpopulations on stem cell properties.

### TgN(hGFAP-GFP) expression and cell lineage commitment

Over time, we observed a loss of GFP-positive cells from 33.5±3.8% in P2 to 17.6±3.9% in P6 during cell propagation (Fig. 1). Under EGF treatment, multipotent stem cells are the most rapidly dividing neural cell population *in vivo* (transient amplifying C cells) [41]. Therefore, under the applied *in vitro* conditions, the GFP-positive cells were a more mature (glial progenitors) or less mature (NSCs) slower-cycling cell population that was overgrown by fast-cycling cells. FACS sorting of NPCs from TgN(hGFAP-GFP/mPLP-DsRed) mice for GFP demonstrated good enrichment in both populations. After one passage, the fraction of GFP-positive NPCs increased from 18% to 87% in the positive-sorted population and that of GFP-negative NPCs increased from 81% to 98% in the negative-sorted population. After 2 weeks of neuronal differentiation, GFP-negative sorted NPCs, in contrast to non-sorted and GFP-positive sorted NPCs, demonstrated a high proportion of neuronal differentiation and proved to be functional *in vitro*. Electrophysiological data revealed essential membrane properties of neurons, i.e., expression of functional voltage-gated ion channels, thus suggesting neuronal development. In contrast, GFP-positive NPCs showed significant less often an expression of the common neuronal marker MAP2 and significant more often an expression of the glial marker GFAP, in addition to signs of senescence. Hence, we assume that GFP expression under the control of the GFAP promoter in our cells more likely indicates commitment to glial fate rather than membership in a NSC subpopulation such as radial glial cells.

## Conclusions

Intraventricularly transplanted Q-NPCs showed excellent survival and integration into the brain after 42 days but did not show any indicator of differentiation other than expression of DCX. Q-NPCs were easily traced by CFP expression but did not demonstrate YFP (Thy1.2), GFP (GFAP) or DsRed (PLP) expression. *In vitro*, besides CFP only GFP(GFAP) was expressed in a subpopulation of cells that were most likely glial progenitors. Thus, Q-NPCs could not be used to visualize NPC differentiation *in vivo*. Nevertheless, Tg(hGFAP-GFP) NPCs represent a promising tool to separate neuronal progenitors from glial progenitors for basic research and transplantation studies.
